# Tetrel Bonding as a Vehicle for Strong and Selective Anion Binding

**DOI:** 10.3390/molecules23051147

**Published:** 2018-05-11

**Authors:** Steve Scheiner

**Affiliations:** Department of Chemistry and Biochemistry, Utah State University, Logan, UT 84322-0300, USA; steve.scheiner@usu.edu; Tel.: +1-435-797-7419

**Keywords:** bipodal, Gibbs free energy, imidazolium, triazolium, counterion, deformation energy

## Abstract

Tetrel atoms T (T = Si, Ge, Sn, and Pb) can engage in very strong noncovalent interactions with nucleophiles, which are commonly referred to as tetrel bonds. The ability of such bonds to bind various anions is assessed with a goal of designing an optimal receptor. The Sn atom seems to form the strongest bonds within the tetrel family. It is most effective in the context of a -SnF_3_ group and a further enhancement is observed when a positive charge is placed on the receptor. Connection of the -SnF_3_ group to either an imidazolium or triazolium provides a strong halide receptor, which can be improved if its point of attachment is changed from the C to an N atom of either ring. Aromaticity of the ring offers no advantage nor is a cyclic system superior to a simple alkyl amine of any chain length. Placing a pair of -SnF_3_ groups on a single molecule to form a bipodal dicationic receptor with two tetrel bonds enhances the binding, but falls short of a simple doubling. These two tetrel groups can be placed on opposite ends of an alkyl diamine chain of any length although SnF_3_^+^NH_2_(CH_2_)_n_NH_2_SnF_3_^+^ with *n* between 2 and 4 seems to offer the strongest halide binding. Of the various anions tested, OH^−^ binds most strongly: OH^−^ > F^−^ > Cl^−^ > Br^−^ > I^−^. The binding energy of the larger NO_3_^−^ and HCO_3_^−^ anions is more dependent upon the charge of the receptor. This pattern translates into very strong selectivity of binding one anion over another. The tetrel-bonding receptors bind far more strongly to each anion than an equivalent number of K^+^ counterions, which leads to equilibrium ratios in favor of the former of many orders of magnitude.

## 1. Introduction

The detection, extraction, and transport of anions is an important element in a wide range of biological and chemical processes [1]. Biological evolution has developed a score of anion binding proteins usually with high selectivity. The sulphate-binding protein of Salmonella typhimurium [2] is an example of one that binds this anion via a number of H-bonds. Another protein is responsible for the binding and transport of phosphate [3] with very high specificity. Still another protein, present in blue-green algae, is highly specific for the nitrate anion [4] and another binds specifically to bicarbonate [5]. While the evolutionary process has developed some very specific and selective anion binding agents, modern technology lags behind. Many receptors make use of general electrostatic interactions and sometimes of H-bonds [6,7,8,9,10,11,12]. The thiourea molecule, for example, is a widely used [13,14,15] anion binder that takes advantage of its H-bonding capability. The guanidinium cation and its derivatives [16,17] have also found use in this regard. However, the anion receptors that have been developed to date still suffer from certain disadvantages. Their selectivity is not optimal and they are unable to detect the presence of a particular anion below a given concentration threshold. Furthermore, at this point in time, the biggest need is the development of highly selective receptors that can function in an aqueous rather than organic or biological environment. “Examples of receptors that are neutral or of low charge and operate in organic–aqueous mixtures are uncommon and those that function in 100% water are rarer still” [1].

One major advancement in this field arose from the growing recognition of the phenomenon of halogen bonds (XBs) [18,19,20,21,22,23,24] where an attractive force occurs between a halogen atom and an electron donor such as the lone pair of an amine. It was not long before researchers applied this concept in order to develop receptors that are highly selective for one anion over another [25,26,27,28,29,30,31,32]. The Beer group [33] found that substitution of H by Br enabled the consequent halogen bond to more effectively bind chloride and that receptors of this type could recognize [34] both chloride and bromide ions purely by virtue of XBs [35,36]. Chudzinski et al. [37] obtained quantitative estimates of the contribution of halogen bonding to the binding of anions to bipodal receptors and later [38] applied halogen bonds to develop pre-organized multi-dentate receptors capable of high-affinity anion recognition. Halogen bonding exerts selectivity for bromide over chloride or other anions in a set of tripodal receptors [39]. Our own group [40,41,42,43,44,45,46] has applied quantum chemical calculations toward solving this issue, which shows that the replacement of H in a series of H-bonding bidentate receptors by halogen atoms can influence their binding to halides. The work detailed a remarkable enhancement of both binding and selectivity especially when the H atom is replaced by I.

A rapidly burgeoning group of studies has extended the basic concepts of the XB to other atoms in the periodic table. Depending upon the particular family to which the atom in question belongs, these bonds have come to be known as chalcogen and pnicogen bonds [47,48,49,50,51,52,53,54,55,56,57,58,59,60]. Given the similarities, it was not surprising that noncovalent bonds of this sort can be every bit as useful as XBs in the context of anion binding and transport, which is being demonstrated in recent work [61,62,63,64]. Tetrel atoms (C, Si, Ge, Sn, Pb) seem capable of engaging in very similar interactions as well, which is becoming increasingly clear [65,66,67,68,69,70,71,72,73]. Therefore, there is every reason to believe that tetrel bonds might find a place in this constellation of noncovalent bonds that can function as integral components in anion receptors.

It was just this idea that motivated our group to recently perform calculations to examine how the latter type of bonds might compare with chalcogens in this context. A set of bidentate receptors, modeled closely after those in a prior experimental study, was constructed [43], which varied in whether the atoms that engaged directly with the anion were chalcogen, pnicogen, or tetrel. The transition from chalcogen to pnicogen to tetrel yielded not only progressively stronger binding to anions but also greater selectivity. In a quantitative sense, the binding energy of halides to a Ge-bonding bidentate receptor was as high as 63 kcal/mol and preferentially bound F^−^ over other halides with a selectivity of 27 orders of magnitude. These quantities are especially impressive since the receptor was electrically neutral, which forgoes the positive charge on many other such candidates. A follow-up study [46] delved somewhat more deeply into this issue by adding halogen atoms to the mix as well and by using a different bidentate receptor structure. It was found that with respect to Cl^−^ and Br^−^, the binding is insensitive to the nature of the binding atom, viz H, halogen, chalcogen, or tetrel. However, there is a great deal of differentiation with respect to F^−^ where the order varies as tetrel > H ~ pnicogen > halogen > chalcogen. The replacement of the various binding atoms by their analogues in the next row of the periodic table enhances the fluoride binding energy by 22% to 56%. The strongest fluoride binding agents utilize the tetrel bonds of the Sn atom while it is I-halogen bonds that are preferred for Cl^−^ and Br^−^.

At this point then, there are sound reasons to believe that tetrel bonding offers a unique opportunity in the design of effective and highly selective receptors. However, there are a number of very important issues that remain to be resolved. In the first place, most of the prior calculations have been centered in the gas phase while it is in solution, especially in water, there is more urgent need for these receptors. Particularly when one is dealing with charged species, the effects of hydration can be expected to be especially strong, so in vacuo trends cannot be simply transferred to water but must be assessed directly. For example, hydration would stabilize the receptor/anion complex but would more heavily stabilize the separate individually charged species. Therefore, gas-phase trends may be radically different in water. It is for this reason that the calculations reported in this study are conducted in an aqueous environment.

Within the realm of tetrel bonds, there is a question as to which tetrel (T) atom would be most effective. Past work has suggested that tetrel bonding is strengthened when the T atom is enlarged, but this phenomenon relates to the gas phase and has not been thoroughly tested in water. The same question pertains to the finding that tetrel bonding is enhanced by electron-withdrawing substituents. How might the strength of tetrel bonding be affected if the tetravalent -TH_3_ group is perfluorinated in water or likewise if the group possesses a positive charge? In a similar vein, most of the bipodal receptors that have come under experimental scrutiny are dications so it is important to assess how a double positive charge affects the binding. Within the context of the construction of the full receptor, the binding group has typically been placed by experimentalists on an imidazolium or triazolium group. Calculations can be used to compare and contrast a wider range of different groups and consider whether the aromaticity of this group is important or whether it even needs to be cyclic. One can address specificity by comparing the binding energetics of a number of various anions to each candidate tetrel-binding receptor. Lastly, since the extraction of an anion from solution by any receptor must overcome the attraction of this anion to counter-ions, this competition must be considered as well.

## 2. Systems and Methods

In the first set of tests, tetrel T atoms examined included the full {Si, Ge, Sn, Pb} set. These were placed into both a -TH_3_ setting and its perfluorinated -TF_3_ counterpart. One of the most commonly used groups to which anion-binding agents have been attached in the past is the imidazole species [9,27,33,34,39,42,74,75,76,77,78] so it is this group that is considered in the pilot set of calculations. Both TH_3_ and TF_3_ were, therefore, affixed to an imidazole moiety and comparisons were made to the same system after protonation of the ring to an imidazolium group. The primary anion used to test binding was Cl^−^, which is representative of the entire halide set without the complications noted earlier for the smaller F^−^, which was prone to engage in asymmetric covalent bonding with the receptor. Another reason for selecting chloride as the prototype anion is the close correspondence observed recently [79] between its calculated binding energy with a series of Lewis acids and the experimental trends arising from NMR measurements. Since this first battery of tests pointed to Sn as the most effective tetrel-bonding atom, it was the focus of the next testbed of calculations, which evaluated a wide range of groups that might replace imidazolium and perhaps enhance the anion binding. These replacements included both aromatic and nonaromatic, cyclic and noncyclic, and both mono and dictations. Having established one or two prime candidates, calculations then turned to comparisons between different anions of chemical and biochemical importance including all four halides, OH^−^, NO_3_^−^, and HCO_3_^−^. Since the receptor must be capable of pulling the anion of choice out of solution where it is closely associated with positive counter-ions, the receptor/anion binding was compared to that with K^+^ cations as model counter-ions.

Calculations were carried out with the Gaussian-09 [80] set of programs. The M06-2X DFT functional [81] was used along with the aug-cc-pVDZ basis set. For the heavy atoms I, Pb, and Sn. The aug-cc-pVDZ-PP pseudopotential was taken from the EMSL library [82,83] so as to incorporate relativistic effects. This level of theory is appropriate for this task as evident by previous work by others [84,85,86,87,88,89] as well as by ourselves in dealing with very similar sorts of systems [40,41,42,90]. The geometries of the receptors and complexes were fully optimized with no restriction, which was assured as minima by the absence of imaginary vibrational frequencies. The binding energy of each anion with its receptor was calculated as the difference between the energy of the complex and the sum of the energies of separately optimized monomers. It was then corrected for basis set superposition error by the counterpoise [91,92] procedure. Gibbs free energies of each complexation reaction are computed at 298 K. To account for solvent effects, the polarizable conductor calculation model (CPCM) was applied [93] with water as the solvent. This approach treats the surroundings as a polarizable continuum with dielectric constant of 78 but does not include explicit water molecules.

## 3. Results

### 3.1. Receptors Containing Imidazole

The binding energies obtained when the Cl^−^ anion was allowed to interact with each of the various tetrel-containing species are reported in Table 1. Several trends are immediately apparent. These quantities are much larger for the cations than for the neutrals, which is sensible in light of the ion-ion interaction in the case of the former. The replacement of the three H atoms on the tetrel atom by F causes a large enhancement as much as six-fold. This increase is especially large for the two heavier tetrel atoms Sn and Pb. Actually, it is the latter two tetrel atoms that consistently show the strongest binding with Sn having a slight edge.

**Figure 1:**
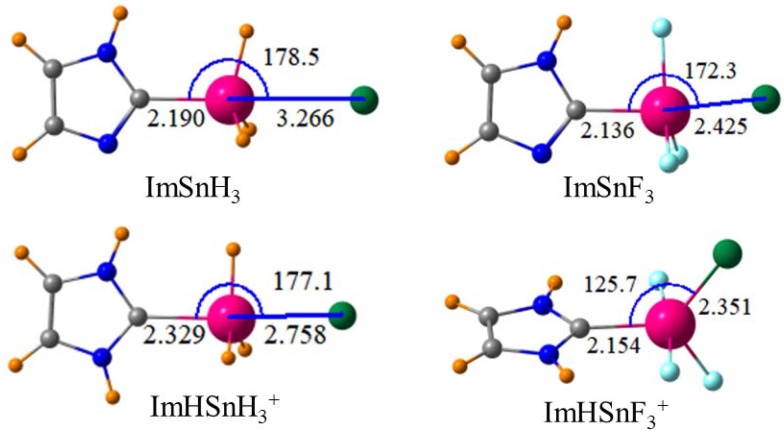
Geometries of indicated Lewis acids with Cl^−^. Distances in Å, angles in degs.

Representative geometries are depicted in Figure 1 for the Sn systems and show trends that parallel the energetics. The R(Sn·Cl) distances are shorter for the cationic receptors and are also shortened when the SnH_3_ group is changed to SnF_3_. Table 2 collects the R(T·Cl) distances for a full range of these complexes. As one would expect from the trends in the energetics, this distance is much shorter for the cations than for the neutral entities and the TF_3_ systems hold the Cl in closer than does TH_3_. The comparisons among the various tetrel atoms are more important. As the tetrel atom grows larger, one would expect a corresponding elongation of R(T·Cl). However, this trend would be opposed by the growing strength of the tetrel bond in the order Si < Ge << Pb < Sn so the pattern is not obvious to predict. Furthermore, there is little relation between tetrel atoms and R for the neutral ImTH_3_ while R gets longer with heavier tetrel atom for TF_3_. The conflict between the two trends is more complicated for the cations. The longest distance occurs for Ge for the TH_3_ systems while there is a clearer trend of longer distances for larger T atoms for TF_3_.

There are several other interesting aspects of the geometry surrounding the T atom. Considering the SnH_3_ systems on the left side of Figure 1, the θ(CSnH) angle in the neutral monomer is equal to 107.8°, which is nearly tetrahedral. It is reduced to 103.1° in the complex with Cl^−^, i.e., the SnH_3_ group flattens toward a trigonal bipyramid. This same group is already fairly flat in the cationic monomer with θ = 102.4°. However, upon complexation with Cl^−^, the SnF_3_ group undergoes a more radical change. Instead of adopting a position nearly opposite the C atom, the Cl moves well out of the imidazolium plane with θ(CSn·Cl) = 125.7°. Note that the R(Sn-Cl) distance of 2.351 Å is only 0.2 Å longer than the R(Sn-C) distance of 2.154 Å. The geometry around the Sn might fairly be described as a trigonal bipyramid with two apical F atoms and with Cl, C, and the third F occupying the three equatorial positions. This set of geometrical parameters and larger scale rearrangement for the ImHTF_3_^+^ complex is not limited to Sn but is characteristic of all four tetrel atoms.

More important than the binding energy itself is the free energy for the complexation reactions. ∆G contains not only zero point and thermal corrections but also entropic contributions. In part as a result of the transition from a pair of subunits to a single complex, the values of ∆G in Table 3 are less negative than ∆E in Table 1 and are even becoming positive in a number of instances. Some of the trends in ∆E survive the additional terms. For example, binding to the cationic receptors is stronger than to the neutrals and the replacement of TH_3_ by TF_3_ bolsters the strength of the interaction. The strongest binding occurs in all cases for Sn and Pb with a slight edge for the former. It might be noted that even within the confines of the strong dielectric environment of water, the binding of Cl^−^ to the cationic ImHTF_3_^+^ species is a minimum of 15 kcal/mol and it rises to more than twice this amount for T=Sn and Pb. As mentioned above, the treatment of solvation here does not include specific interactions between the solvated system and discreet water molecules. The inclusion of this might have a bearing on these results.

### 3.2. More General Receptors

**Figure 2:**
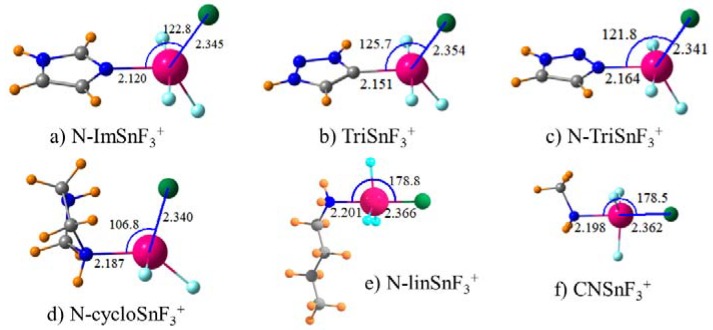
Geometries of indicated Lewis acids with Cl^−^. Distances in Å, angles in degs.

The success of cationic ImHSnF_3_^+^ as a receptor can be used as a starting point to explore modifications that might further enhance the binding. In the first place, one can imagine the ImHSnF_3_^+^ group being attached not to one of the imidazole C atoms but rather to N. The more electron-withdrawing power of the latter might strengthen the ability of the Sn atom to attract a nucleophile. Formation of this complex, pictured in Figure 2a, does enhance the binding by some 5 kcal/mol, which is indicated in Table 4. The geometry is basically unaltered by this change besides a small contraction of the R(Sn·Cl) distance. A second modification would be to add a third N atom to imidazolium to generate a triazolium species, which is shown in Figure 2b. It is this group which has served as the point of attachment for the anion-binding species in a number of experimental works [7,27,28,29,30,35,36,62,94,95,96,97,98,99]. Table 4 indicates that this change weakens the interaction by roughly 10%. On the other hand, switching the point of connection from C to N again raises ∆G to a point where it surpasses that of N-Im by a small amount with R(Sn·Cl) reduced to 2.341 Å, which is seen in Figure 2c.

Since the receptors considered at this point all contain a heteroaromatic ring, the question arises as to the importance of this aromaticity to the binding. The five-membered imidazole ring was, therefore, fully saturated with H atoms, which leads to a nonaromatic ring by retaining the two N atoms. This loss of aromaticity does not reduce the chloride affinity. When attached to an N atom of the CH_2_CH_2_NHCH_2_NH^+^ ring (abbreviated as N-cyclo), the SnF_3_ group in Figure 2d binds Cl^−^ with approximately the same ∆G as the aromatic N-ImHSnF_3_^+^ counterpart in Figure 2a. Additionally, there is a slight enhancement in ∆E. This result begs the question as to whether the cyclic nature of the receptor is an important component at all or whether the second N atom is essential. The heterocycle of Figure 2d was, therefore, replaced with a simple amine CH_3_(CH_2_)_3_NH_2_ with the same number of five heavy atoms, which is represented in Figure 2e. This species, abbreviated as N-linHSnF_3_^+^, suffers only a very small loss of binding energy with ∆G still exceeding 40 kcal/mol. One may note a change in geometry around the Sn atom. In this simple amine, the Cl atom situates itself directly opposite the N atom, which leaves the three F atoms in equatorial sites. The next question relates to the length of the amine. If it is shortened from a *n*-butyl chain to a simple methyl group, how might that affect the binding. Such a shortening yields a small enhancement in the chloride affinity, which raises ∆G from 40.85 kcal/mol to 42.57 kcal/mol, which is indicated by the CNSnF_3_^+^ entry in Table 4 with the corresponding complex illustrated in Figure 2f.

**Figure 3:**
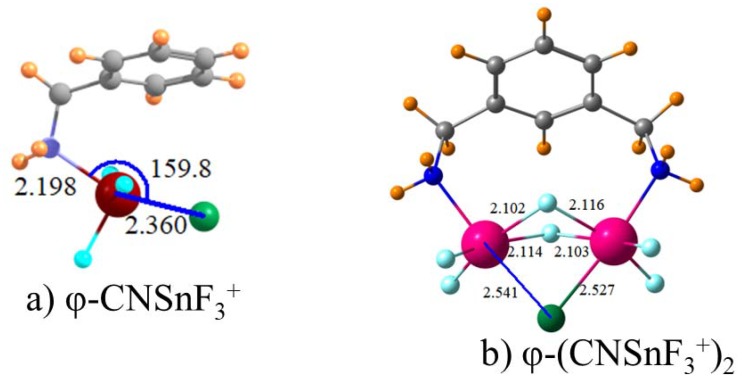
Geometries of indicated Lewis acids with Cl^−^. Distances in Å, angles in degs.

There may be a particular advantage in the placement of the receptor on an aromatic system such as a phenyl ring, which is typical of those that have been considered experimentally and computationally in the past. In order to address this issue, the methylamine molecule was covalently attached to a phenyl group, which is illustrated in Figure 3a. Instead of augmenting the binding, this attachment had the opposite effect of reducing the binding energy by about 4 kcal/mol, or 10%, which was noted by the φ-CNSnF_3_^+^ entry in Table 4. It may be noted that this attachment to the phenyl ring induces a change in the geometry wherein the θ(NSn·Cl) angle decreases by 19° from 178.5° to 159.8° although the R(Sn·Cl) distance remains virtually unchanged. In addition of the attachment to a spacer such as a phenyl group, the commonly used receptors contain a pair of binding units in a bidentate arrangement. This sort of structure was mimicked by connecting two CH_2_NH_2_SnF_3_^+^ groups onto the same benzene ring. As illustrated in Figure 3b, the chloride ion occupies a near symmetric position, which is bound to both Sn atoms. Additionally, one F atom from each of the SnF_3_ groups swings around so that they too are symmetrically disposed to the two Sn atoms. As indicated in Table 4, this bidentate receptor represents an enhancement in the binding with ∆G increasing from 38 kcal/mol to 46 kcal/mol. On the other hand, given the doubling of the positive charge on this receptor and the addition of a second tetrel bond, this 20% increase is a rather disappointing increment. Another reason for disappointment is that the bidentate geometry in Figure 3b has a more linear θ(NSn·F) angle of 173° compared to 160° in Figure 3a, which would ordinarily be more conducive to a strong noncovalent bond.

**Figure 4:**
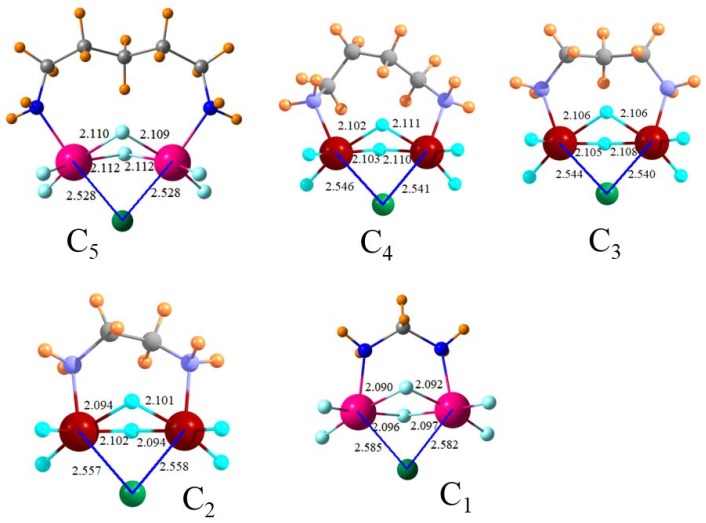
Geometries of cyclic F_3_SnNH_2_(CH_2_)_n_NH_2_SnF_3_ dications (C_n_) with Cl^−^. Distances in Å, angles in degrees.

Since aromaticity offers little advantage, there is little reason to connect the two SnF_3_^+^ groups through a phenyl ring spacer. Perhaps a bidentate arrangement in the same spirit could be offered by a simple set of methylene groups as an alkyl diamine. These systems were designed with varying numbers *n* of methylene groups in SnF_3_^+^NH_2_(CH_2_)_n_NH_2_SnF_3_^+^, permitted to react with Cl^−^ and the resulting structures are depicted in Figure 4. All have the desired bipodal binding with the Cl nearly symmetrically disposed toward the two Sn atoms and with similar R(Sn·Cl) distances. The energetic data in Table 4 indicates that all of these receptors bind more strongly to Cl^−^ than does the original receptor φ-(CNSnF_3_^+^)_2_, i.e., the benzene connector offers no advantage. Of the various size diamine dications, C_5_ and C_1_ are the least favorable and C_4_ and C_2_ the most favorable.

**Figure 5:**
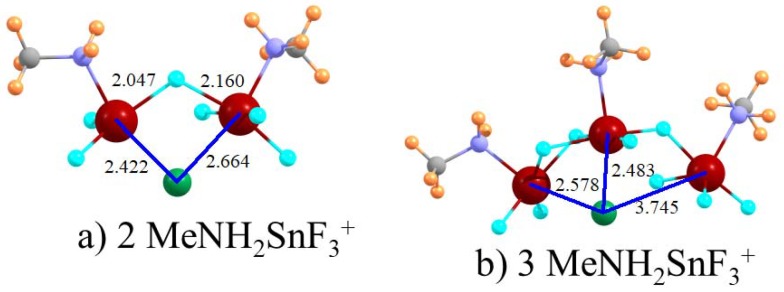
Geometries of Cl^−^ with (**a**) 2 and (**b**) 3 CH_3_NH_2_SnF_3_^+^ cations. Distances in Å.

The structure of each receptor in Figure 3b and Figure 4, with its bidentate binding to the chloride, may impair the ability of each of the two tetrel bonds from achieving its full potential interaction energy. For example, the θ(NSn·Cl) bond would naturally incline toward 180° but this is not possible for a number of these complexes. In order to relieve this geometrical stress, the two cations within the single molecule were separated into a pair of mono-cations. In particular, the Cl^−^ was allowed to bind to two individual MeNH_2_SnF_3_^+^ ions and the resulting complex is pictured in Figure 5a. Despite the geometrical freedom, the two tetrel-bonding groups adopt a geometry very much like the single-molecule dications. Specifically, the two θ(NSn·Cl) angles in Figure 5a are 166° and 157°, which is somewhat deviant from linearity. These angles are not very different from those in the C3 and C4 diamines with angles of 157° and 160°, respectively. Additionally, perhaps more to the point, the freedom granted by the pair of mono-cations does not enhance the binding energy. Table 4 shows that ∆G is 53.5 kcal/mol, which is even lower than for most of the diamine dications (although ∆E does profit from a small enhancement). As a last point of interest in this regard, the addition of a third MeNH_2_SnF_3_^+^ mono-cation increases the chloride binding but only by a small degree of 24%. This small increase may be due to steric crowding involving the third tetrel group. As evident in Figure 5b, the third R(Sn·Cl) distance is 3.745 Å, which is more than a full Å longer than the other two distances. The close proximity of the tetrel groups in Figure 5 was not imposed since optimizations were begun with these groups were nearly opposite one another.

### 3.3. Anions Other than Chloride

The forgoing analysis has been based on Cl^−^ as the universal anion. However, one of the important roles of a desirable anion receptor is its ability to distinguish among a sea of different anions. For this purpose, MeNH_2_SnF_3_^+^ was chosen as the prototype monocationic receptor and the C_2_ diamine SnF_3_^+^NH_2_(CH_2_)_2_NH_2_SnF_3_^+^ as dication. Both of these exhibit strong binding to the chloride. In addition to the four simple halides, other anions chosen for examination, due to their importance and prevalence, are OH^−^, NO_3_^−^, and HCO_3_^−^.

The binding energetics collected in Table 5 indicate that OH^−^ engages in the strongest interactions with either of the cationic receptors. In the case of the monocation, OH^−^ is followed by F^−^ and then by HCO_3_^−^. The latter two anions reverse places for the dication. There is little to distinguish NO_3_^−^ from the three larger halides whose binding follows the order of increasing size: Cl^−^ > Br^−^ > I^−^. As was observed in the earlier cases, ∆E is a bit more negative than ∆G.

**Figure 6:**
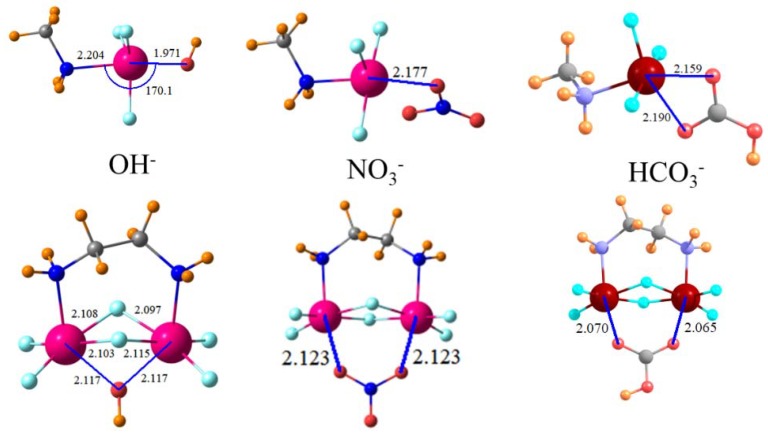
Geometries of indicated anion with CH_3_NH_2_SnF_3_^+^ cation in upper half and F_3_SnNH_2_(CH_2_)_2_NH_2_SnF_3_^+2^ dication in lower half. Distances in Å.

The geometries of the various complexes with the halides are parallel to those for Cl^−^. The same may be said of the structures involving OH^−^, which can be seen in Figure 6. It is the O atoms of NO_3_^−^ and HCO_3_^−^ that directly interact with Sn and both are able to engage in bifurcated tetrel bonding with more than one O atom participating. Nonetheless, despite this possible advantage, it is the OH^−^ anion with its single O atom that is most strongly bound.

### 3.4. Comparison of Receptors with Mobile Counterions

In order to extract any anion from solution, a receptor must compete with the anion’s counterions. K^+^ was chosen as a typical counter-ion, which might commonly occur. The concentration of the positive charge on a single atom might be anticipated to forge a very strong ion-ion interaction with each of the anions mentioned above. However, the binding energetics are comparatively quite small, which may be seen in Table 6. For example, Cl^−^ binds to K^+^ with a ∆G of only −1.7 kcal/mol compared to the very much larger −42.6 kcal/mol for the tetrel-bonding MeNH_2_SnF_3_^+^. Overall, the latter binds more strongly to the various anions than does K^+^ by a factor between 10 and 40. The addition of a second K^+^ can be used to compare with the dications. As seen in Table 6, this binding energy is no more than 10 kcal/mol, which compares with quantities between 60 kcal/mol and 113 kcal/mol for the dual tetrel bonded systems in Table 5.

These energy differences translate into a tremendous advantage for the tetrel-bonding species over the simple K^+^ cations in the capture of these anions. If one expresses this advantage as the equilibrium constant K = exp(δ∆G/RT) where δ∆G represents the difference in binding free energy between the former and the corresponding number of K^+^ cations. The values obtained are listed in Table 7 at 25 °C. These advantages are very large from a minimum of 10^27^ all the way up to 10^75^. Additionally, the dicationic receptors display a much larger advantage than the mono-cations.

### 3.5. Geometric Deformations of Monomers

It has been observed before [100,101] that substituents surrounding tetravalent tetrel atoms hinder the unimpeded approach of a nucleophile. If some of the substituents are bulky enough, they may prevent the formation of a tetrel bond entirely. However, even when smaller substituents are present, they must be pulled away to make room for the approaching nucleophile, which induces a certain amount of deformation energy into the Lewis acid molecule. This quantity has been shown to be as large as 20 kcal/mol and can be even larger [100] than the binding energy itself. This situation occurs in the tetrel-bonded complexes here as well. From the diagrams of the various complexes, one can see that the geometry changes around the tetrel atom are not a mere flattening out of the SnF_3_ group to accommodate the chloride. It is true that the structure around the Sn atom adjusts from tetrahedral in the monomer to something more akin to a trigonal bipyramid within the complex. However, the apices of this bipyramid are not necessarily the C/N atom of the receptor and the Cl. In many of the optimized structures, these two atoms adopt equatorial positions along with one of the F atoms while the two remaining F atoms are positioned at the apices. 

The deformation energies of the various cationic Lewis acids caused by their complexation with Cl^−^ are reported in the second column of Table 8 where it may be seen that there is a larger deformation energy for the first five mono-cations, in which all undergo the greater distortion required to rearrange so as to place F atoms at the apices. The deformation energies of the latter complexes all exceed 30 kcal/mol while the simpler rearrangements that retain the three F atoms in equatorial positions lie between 24 kcal/mol and 27 kcal/mol.

Rearrangements of the bipodal receptors are a bit simpler conceptually. The monomers contain a pair of Sn-F-Sn bridges not unlike the structures of the complexes pictured in the various figures. Therefore, the bulk of the rearrangement involves that necessary to make the two θ(N-Sn-Cl) angles as close to linearity as possible. In the φ-(CNSnF_3_^+^)_2_ dication complex, for example, this angle differs from linearity by only 6°, which involves a deformation that requires 45 kcal/mol. The C_5_ diamine achieves an 8° nonlinearity at a cost of only 38 kcal/mol, which suggests a bit more flexibility. The smaller diamines require a bit less deformation energy even if sacrificing greater nonlinearity: θ(N-Sn-Cl) = 20°, 23°, 34°, and 45°, respectively, for C_4_, C_3_, C_2_, and C_1_ diamines. Note that the binding energy in the first column of Table 8 does not suffer from this increasing nonlinearity.

When the deformation energy is added to the total binding energy ∆E, the resulting E_int_ represents the interaction between Cl^−^ and the Lewis acid, once it has been deformed to the geometry, it adopts within the context of the full dimer. These quantities in the last column of Table 8 are quite large. They lie in the range of 73 kcal/mol to 93 kcal/mol for the mono cations especially large for N-ImSnF_3_^+^ wherein the SnF_3_ group is attached to the N atom of imidazolium. E_int_ is even larger for the dications where it hovers consistently around 100 kcal/mol. Note that the interaction energy of Cl^−^ with a pre-deformed dicationic chelator, like the binding energy, remains quite a bit smaller than twice the analogous quantity for the monocations. These tetrel bond energies cannot be considered as simply additive.

## 4. Discussion

Of the various tetrel atoms tested, Sn forms the strongest interactions with a chloride anion, which is followed closely by Pb. The tetrel bond is strongly enhanced when the TH_3_ group is perfluorinated to TF_3_. The interaction is further strengthened if the molecule containing the tetrel atom is endowed with a full positive charge. With this information as a starting point, the imidazolium group to which the SnF_3_ group is attached was varied in a methodical way to see if there were any ways to improve the binding. Binding is improved if this group is covalently attached to a Nitrogen atom of imidazolium rather than Carbon. On the other hand, replacement of imidazolium by triazolium had a slight weakening effect even though the tetrel bond is enhanced if the point of attachment is changed from C to N. The aromaticity of either of these two groups seems irrelevant since the replacement of imidazolium by its fully saturated five-membered ring analogue has no deleterious effect on the tetrel bond. Nor is the cyclic structure important, the binding is scarcely affected when a linear chain is used instead. The length of this chain on the N atom connection to SnF_3_ is unimportant as well since its shortening from *n*-butyl to a simple methyl group produces only a small enhancement. There seems little advantage in placing this amine group on a phenyl connector since doing so weakens the tetrel bond by perhaps 10%.

A chelating arrangement whereby the Cl^−^ forms tetrel bonds to two SnF_3_ groups simultaneously increases the total binding energy but by far less than a factor of two. For example, placing two CH_3_NH_2_SnF_3_^+^ groups on the phenyl ring produces only a magnification of the total binding energy by 21% when compared to that of a single such tetrel-bonding group. The size of this increase is not a result of geometric distortion since both θ(NSn·Cl) angles lie within 6° of linearity within this clathrate structure. Replacing the rigid phenyl ring by a more flexible (CH_2_)_n_ alkane chain improves the overall binding regardless of the length of this chain. The optimal length appears to be *n* = 2. Placing the two SnF_3_ groups onto two separate molecules does not result in a stronger interaction, which suggests that steric constraints within the single dication molecule are not a detrimental factor. Just as adding a second group resulted in a magnification of only 1.2, a third such cation increases the binding free energy by the same factor. The modesty of the enhancement arising from a doubling of the positive charge on the receptor echoes recent [99,102] experimental findings.

It is worth reiterating that a very recent work [79] suggested that Cl^−^ is an excellent choice as the test anion since its calculated binding to a series of Lewis acids mimics the experimental trends arising from NMR measurements. While the binding of Cl^−^ is just a bit stronger than the larger halides as well as NO_3_^−^, HCO_3_^−^ binds more strongly to the MeNH_2_SnF_3_^+^ monocation. The smaller size of F^−^ with its concentrated negative charge leads to a larger binding free energy and OH^−^ even more so. The calculated trend of diminishing binding that accompanies the increasing size of the halide is consistent with experimental findings [99]. These same trends are in evidence when these anions engage in a bifurcated tetrel bond with a uni-molecular SnF_3_^+^NH_2_(CH_2_)_2_NH_2_SnF_3_^+^ dication even though the magnitudes are larger. These differences in binding energy can result in highly selective receptors. For example, the 24 kcal/mol difference in ∆G binding of F^−^ over Cl^−^ in Table 5 translates to a 10^17^ equilibrium preference of the former over the latter. Even smaller differences in ∆G reflect substantial selectivity. The 3.4 kcal/mol advantage of Cl^−^ over Br^−^ yields a 300-fold equilibrium ratio. However, the very strong binding of OH^−^ might preclude the use of these receptors in basic environments where hydroxide would likely displace other anions.

In order to preferentially bind with an anion in solution, a receptor must successfully compete with counterions. The tetrel-bonding receptors examined here are extremely effective in this regard. Their binding energies with the various monoanions are much greater than those of K^+^ counterions despite the ability of the latter to move freely around each anion. The preference of any given anion for the monocationic tetrel-bonding receptors, over a K^+^ counterion, expressed as an equilibrium ratio, varies between 10^27^–10^53^. This preference is even larger for the dications when compared to a pair of K^+^ counterions, which rise up to as high as 10^75^.

It will be observed that both Gibbs free energy (∆G) and electronic energy (∆E) has been provided for all of the complexation reactions here. The former corrects the latter for zero-point vibrational energies as well as entropic effects. The latter additions make ∆G less negative than ∆E, but the discrepancy is fairly uniform and is typically on the order of 7–10 kcal/mol, which is a bit larger for the dications. As an end result, both energetic quantities obey similar trends.

It should be stressed that the self-consistent reaction field approach used here to model immersion in a solvent represents only an approximation of the full solvation effects. This model treats the solvent as a dielectric continuum that reacts to, and stabilizes, the charge distribution within the solute in an iterative manner. In doing so, it essentially averages over the many configurations that the solvent molecules will adopt over the course of a measurement. However, specific interactions of any individual solvent molecule with the solute are not explicitly evaluated. For this reason, the calculated energetics in water should be treated as only approximations. Nonetheless, this procedure has the virtue of providing some measure of the relative stabilization caused by immersing the solute in the solvent milieu. The trends in the data that emerge are likely realistic and differences from one system to the next of more than a few kcal/mol can be treated as meaningful. For example, the very large equilibrium ratios in Table 7 between the preference of each anion for a tetrel-bonding receptor vs K^+^ counterions are very unlikely to be reversed if other means of estimating solvation are employed.

Due to the high dielectric constant of water, solvation has quite a large impact on the binding energies. Taking the ImGeTH_3_ complex with Cl^−^ as an example, the interaction energy in water of −1.9 kcal/mol rises to −14.6 kcal/mol in vacuo. The effect on the charged ImGeTF_3_^+^ receptor is even more extreme since ∆E grows from −32.1 kcal/mol to −144.4 kcal/mol. Very similar increases are observed in ∆G. One may also consider how solvation contributes to the huge advantage that the tetrel-bonding receptors enjoy over K^+^ in the competition for an anion. Table 6 indicates a very weak interaction between K^+^ and Cl^−^ in water with ∆G of only −1.72 kcal/mol, which is a major factor in the advantage of the tetrel-bonding receptor in the competition for this anion. The situation in the gas phase leads to much larger binding energies. Without the very substantial solvation energy of the cation, ∆G is greatly enlarged to −113.1 kcal/mol in vacuo. Taking the tetrel-bonding MeNH_2_SnF_3_^+^ cation as a counterpoint, its binding energy with Cl^−^ of −42.57 kcal/mol in water increases to −181.1 kcal/mol in vacuum, which is an even larger increment. As a result, the 41 kcal/mol advantage that MeNH_2_SnF_3_^+^ holds over K^+^ in solution is increased to 68 kcal/mol without the moderating influence of water. Therefore, one may surmise that the stronger binding of tetrel-bonding species when compared to a small and compact counterion is intrinsic and is not an artifact of the solvation phenomena. 

The reason for this reduced advantage in water derives from the solvation energies of the individual species. For exemplary purposes, one may consider the interactions of Cl^−^ with both MeNH_2_SnF_3_^+^ and K^+^. Considering first the monomers, the solvation energy of K^+^ is larger by 9 kcal/mol than that of MeNH_2_SnF_3_^+^ due to its smaller size and more compact charge. A similar advantage accrues to the K^+^∙Cl^−^ ion pair vs. the larger tetrel-bonded complex where it increases by 24 kcal/mol. This greater stabilization advantage of the K^+^∙Cl^−^ complex vs the separate ions increases its binding energy relative to the MeNH_2_SnF_3_^+^ analogue. The net result is that the lesser binding energy of K^+^ vs the tetrel bond in the gas phase is reduced by 15 kcal/mol in water.

It might finally be remarked that some of these interactions between the receptor and the anion are quite strong since they are in excess of 50 kcal/mol. When combined with the rather short R(Sn·X) distances, it would be legitimate to refer to many of these interactions as bordering on covalent with the Sn atom adopting a hyper-valent bonding character. The arrangement of the atoms around the Sn atom in Figure 2, for example, might best be described as pentavalent trigonal bipyramidal. An octahedral hexavalent environment, albeit a distorted one, could be invoked for a number of the bipodal receptors, which is shown in Figure 4.

In conclusion, tetrel bonding offers a highly attractive way of forming strong complexes with anions that can easily extract these anions from an aqueous environment containing counter-ions. The -SnF_3_ group is particularly effective in this regard especially when the receptor contains a positive charge. A bipodal dicationic receptor has advantages over a mono-cation that can engage in only a single tetrel bond. It is hoped that the ideas presented here may guide researchers in the synthesis and testing of improved anion receptors.

## Figures and Tables

**Table 1:** Binding energy (kcal/mol) between Cl^−^ and ImTR_3_ and ImHTR_3_^+^.

	Neutral		Cation	
T	ImTH_3_	ImTF_3_	ImHTH_3_^+^	ImHTF_3_^+^
Si	−1.79	−2.73	−5.74	−23.98
Ge	−1.87	−8.81	−5.26	−32.12
Sn	−3.57	−23.47	−10.14	−43.54
Pb	−2.78	−20.62	−7.87	−39.51

**Table 2:** R(T·Cl) (Å) in optimized complexes.

	Neutral		Cation	
T	ImTH_3_	ImTF_3_	ImHTH_3_^+^	ImHTF_3_^+^
Si	3.441	2.293	2.557	2.112
Ge	3.491	2.310	3.032	2.178
Sn	3.266	2.425	2.758	2.351
Pb	3.435	2.467	2.988	2.419

**Table 3:** ∆G(298 K) (kcal/mol) for interactions between Cl^−^ and ImTR_3_ and ImHTR_3_^+^.

	Neutral		Cation	
	ImTH_3_	ImTF_3_	ImHTH_3_^+^	ImHTF_3_^+^
Si	+4.37	+5.42	+2.12	−14.95
Ge	+4.77	−1.23	+2.32	−23.91
Sn	+2.75	−13.31	−2.77	−36.53
Pb	+3.01	−12.44	−1.73	−32.25

**Table 4:** Energetics (kcal/mol) for interactions between Cl^−^ and indicated receptors.

	∆G	∆E
**Monocations**		
ImSnF_3_^+^	−36.53	−43.54
N-ImSnF_3_^+^	−41.75	−49.99
TriSnF_3_^+^	−33.29	−41.30
N-TriSnF_3_^+^	−42.65	−51.67
N-cycloSnF_3_^+^	−42.39	−51.76
N-linSnF_3_^+^	−40.85	−49.53
CNSnF_3_^+^	−42.57	−50.76
φ-CNSnF_3_^+^	−38.03	−46.43
**Dications**		
φ-(CNSnF_3_^+^)_2_	−46.00	−54.83
C_5_ diamine	−53.63	−62.76
C_4_ diamine	−62.21	−73.11
C_3_ diamine	−60.42	−70.52
C_2_ diamine	−63.30	−71.94
C_1_ diamine	−53.33	−62.73
***n* MeNH_2_SnF_3_^+^**		
*n* = 2	−53.53	−75.13
*n* = 3	−66.38	−103.77

**Table 5:** Energetics (kcal/mol) for interactions between anions and indicated mono and di-cationic receptors.

Anion	∆G	∆E
**MeNH_2_SnF_3_^+^**		
F^−^	−64.54	−73.61
Cl^−^	−42.57	−50.76
Br^−^	−38.83	−46.64
I^−^	−36.46	−43.85
OH^−^	−78.62	−89.61
NO_3_^−^	−38.46	−52.81
HCO_3_^−^	−52.44	−67.65
**C_2_ Diamine Dication: SnF_3_^+^NH_2_(CH_2_)_2_NH_2_SnF_3_^+^**		
F^−^	−87.88	−98.36
Cl^−^	−63.30	−71.94
Br^−^	−59.98	−68.20
I^−^	−58.04	−66.09
OH^−^	−112.98	−126.58
NO_3_^−^	−64.86	−79.14
HCO_3_^−^	−94.46	−109.52

**Table 6:** Energetics (kcal/mol) for interactions between anions and one or two K^+^ cations.

	∆G	∆E
**K^+^**		
F^−^	−6.97	−11.73
Cl^−^	−1.72	−5.95
Br^−^	−0.90	−4.97
I^−^	+0.07	−3.85
OH^−^	−6.18	−12.61
NO_3_^−^	−1.75	−8.88
HCO_3_^−^	−3.85	−10.98
**2 K^+^**		
F^−^	−10.03	−21.71
Cl^−^	−1.04	−10.56
Br^−^	−0.02	−8.73
I^−^	+1.96	−6.60
OH^−^	−10.07	−21.79
NO_3_^−^	−1.95	−15.92
HCO_3_^−^	−5.08	−18.82

**Table 7:** Preference of anions for tetrel-bonding species over one or two K^+^ cations.

Anion	Monocation/K^+^	Dication/2K^+^
F^−^	1.4 × 10^42^	1.0 × 10^57^
Cl^−^	8.2 × 10^29^	3.9 × 10^45^
Br^−^	6.0 × 10^27^	8.2 × 10^43^
I^−^	5.7 × 10^26^	8.7 × 10^43^
OH^−^	1.1 × 10^53^	2.3 × 10^75^
NO_3_^−^	7.7 × 10^26^	1.2 × 10^46^
HCO_3_^−^	3.8 × 10^35^	2.9 × 10^65^

**Table 8:** Binding, deformation, and interaction energy (kcal/mol) for interactions between Cl^−^ and indicated receptors.

Receptor	∆E	E_def_	E_int_^a^
**Monocations**			
ImSnF_3_^+^	−43.54	32.82 ^b^	−76.36
N-ImSnF_3_^+^	−49.99	43.40 ^b^	−93.39
TriSnF_3_^+^	−41.30	34.33 ^b^	−75.63
N-TriSnF_3_^+^	−51.67	30.36 ^b^	−82.03
N-cycloSnF_3_^+^	−51.76	33.30 ^b^	−85.06
N-linSnF_3_^+^	−49.53	24.18	−73.71
CNSnF_3_^+^	−50.76	24.52	−75.28
φ-CNSnF_3_^+^	−46.43	27.15	−73.58
**Dications**			
φ-(CNSnF_3_^+^)_2_	−54.83	44.98	−99.81
C_5_ diamine	−62.76	38.47	−101.23
C_4_ diamine	−73.11	27.88	−100.99
C_3_ diamine	−70.52	31.85	−102.37
C_2_ diamine	−71.94	29.13	−101.07
C_1_ diamine	−62.73	36.88	−99.61

^a^ E_int_ = ∆E − E_def_; ^b^ two F atoms at apices of trigonal bipyramid in complex.
